# Wireless magnetoelectrically powered organic light-emitting diodes

**DOI:** 10.1126/sciadv.adm7613

**Published:** 2024-03-06

**Authors:** Julian F. Butscher, Sabina Hillebrandt, Andreas Mischok, Anna Popczyk, Jonathan H. H. Booth, Malte C. Gather

**Affiliations:** ^1^ Centre of Biophotonics, SUPA, School of Physics and Astronomy, University of St Andrews, St Andrews, UK.; ^2^ Humboldt Centre for Nano- and Biophotonics, Department of Chemistry, University of Cologne, Cologne, Germany.

## Abstract

Compact wireless light sources are a fundamental building block for applications ranging from wireless displays to optical implants. However, their realization remains challenging because of constraints in miniaturization and the integration of power harvesting and light-emission technologies. Here, we introduce a strategy for a compact wirelessly powered light-source that consists of a magnetoelectric transducer serving as power source and substrate and an antiparallel pair of custom-designed organic light-emitting diodes. The devices operate at low-frequency ac magnetic fields (~100 kHz), which has the added benefit of allowing operation multiple centimeters deep inside watery environments. By tuning the device resonance frequency, it is possible to separately address multiple devices, e.g., to produce light of distinct colors, to address individual display pixels, or for clustered operation. By simultaneously offering small size, individual addressing, and compatibility with challenging environments, our devices pave the way for a multitude of applications in wireless displays, deep tissue treatment, sensing, and imaging.

## Teaser

Compact OLEDs fabricated on magnetoelectric transducers enable ever smaller wireless light sources.

## INTRODUCTION

Wireless light sources offer perspectives for novel wirelessly powered displays (*1*) and potent biomedical implants, e.g., for alternative treatment modalities (*2*), advanced sensing (*3*), and in vivo imaging (*4*). Wireless devices can operate in challenging environments where the on-site integration of leads and batteries is difficult or outright impossible. For instance, smart contact lenses offer new opportunities for augmented reality but need to be removable and extremely lightweight (*5*). Similarly, implanted photonic devices, for example, for photodynamic therapy (*6*), optical sensing (*7*), or optogenetic techniques (*8*), need to be light-weight and compact to minimize the burden on animal models (and possibly patients in the future) and to cope with the limited available space.

There exists a range of wirelessly operating devices with varying advantages for specific applications. For many of these, the power receiver is the largest component (often tens of cubic millimeters in volume) and/or must be near the power transducer. In biomedical applications, this means that the receiver must be implanted at shallow depth, usually subcutaneously, and the actual light delivery device—typically a light-emitting diode (LED) or an optical fiber—is then connected via tethers and placed in the region of interest, e.g., connecting through the skull and to a relevant site in the brain of an animal model (*9*–*12*). The connection between receiver and light delivery device then further adds to the footprint and invasiveness of the device. To overcome these issues, there is a need for power receivers that (i) can maintain efficient wireless power transfer over millimeters or even centimeters, even in opaque, scattering, or watery environments, and (ii) allow the direct integration of light sources on them, without adding considerably to their volume and weight.

There are two principal ways for wireless power transfer in an optically opaque environment: mechanical pressure waves in the form of ultrasound and electromagnetic fields. In both cases, lower frequency waves are preferable because of their weaker absorption by most materials, including in biological tissue; a particularly low absorption is observed for electromagnetic fields in the regime below 1 MHz (*13*). However, classical antennas would have to be tens of meters in length to efficiently operate at these frequencies, which is not practical for most applications. While inductive energy transfer is possible at low electromagnetic frequencies, Faraday’s law requires coil diameters of about 1 cm for a frequency of 1 MHz to provide a high enough voltage to power a light source, thus limiting the extent to which this power transfer modality can be miniaturized. Nevertheless, subcentimeter-sized wirelessly powered implantable light sources have been developed in recent years. By integrating an LED inside a helical coil, Bansal *et al.* (*14*) developed a miniaturized 15 mm^3^ light source operated at gigahertz frequency. However, tissue absorption at this operating frequency might represent challenges for device operation. Operating at lower frequency, but with a larger device footprint, are wirelessly powered LEDs that use inductive power transfer at the radio frequency (RF) standard frequency of 13.56 MHz (*15*, *16*). Using ultrasonic powering, Kim *et al.* (*17*) presented a wirelessly powered LED device with a volume of 16 mm^3^ by soldering two LEDs directly onto a piezoelectric cube. The use of ultrasound, however, requires direct contact between ultrasound transducer and tissue to prevent ultrasound reflection. Furthermore, the absorption of ultrasound by tissue can lead to considerable heating.

A promising alternative to achieve small device size at low frequencies is based on the composite magnetoelectric (ME) effect. For example, electrical neuronal stimulation has been demonstrated using ME nanoparticles (*18*) and laminate structures (*19*). Furthermore, ME transducers can serve as sensors (*20*, *21*), antennae (*22*, *23*), and energy harvesters (*24*). Singer *et al.* (*19*) recently showed that it is possible to wirelessly power an inorganic LED soldered to the surface electrodes of a simple bilayer ME transducer. Kang *et al.* and Lu *et al.* (*25*, *26*) used ME energy harvesters to power an entire array of LEDs through active driving as well as harvesting of stray magnetic fields from electric appliances. In this prior work, however, the LEDs were connected to the ME transducers using bulky wires which led to a relatively large system overall.

Organic LEDs (OLEDs), which form the basis of almost all modern smartphone displays, offer several advantages over their inorganic counterparts. OLEDs consist of stacks of several organic layers and typically have an overall thickness of less than 1 μm. They can emit light at any wavelength of the visible spectrum; for instance, to realize the red, green, and blue subpixels in a display or to address a particular photodynamic sensitizer (*27*) or optogenetic channel (*28*). Unlike inorganic LEDs, OLEDs are area emitters and can thus assume any two-dimensional shape, e.g., to match a geometrical constraint imposed by a particular application (*29*). Last, OLEDs can be fabricated on a variety of different substrates, including flexible substrates that conform to the topology of the target of light delivery (*30*, *31*) and on silicon chips that contain sophisticated driver and control electronics (*32*).

In this work, we present a wireless LED that combines an ME transducer as its power source and a tailored OLED stack as the light source. The OLED is deposited directly onto the thin-film ME transducer by thermal evaporation of its different organic materials. This process is readily scalable and ensures that the OLED adds negligible weight and volume to the device. We show that the emission color of the device can be easily tuned by changing the emitter material in the OLED and that the devices can be operated wirelessly even when placed inside a light-scattering aqueous medium at a depth of several centimeters. Furthermore, we address multiple devices separately, without the need for additional on-device electronics, by tuning the driving magnetic field to the resonance frequency of ME transducers with slightly different dimensions. Our study thus signposts a promising path toward a class of wireless ultracompact photonic devices.

## RESULTS

### ME effect, device design, and OLED integration

**Fig. 1.**
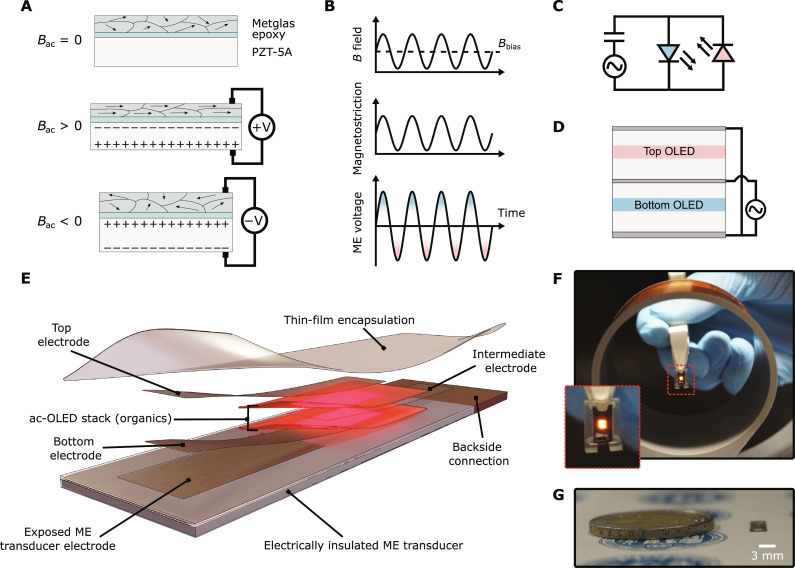
ME effect, device design, and integration of OLED. (**A**) Working principle of ME transducer consisting of a magnetostrictive metal ribbon (Metglas) bonded to a piezoelectric ceramic sheet (PZT) by an epoxy. Exposure to a magnetic field *B*_ac_ leads to deformation of the Metglas and thus induces a voltage across the PZT. (**B**) Schematic showing the evolution of magnetic field (top), magnetostriction (middle), and resulting ME voltage (bottom) over time. Because of the isotropic expansion behavior of magnetostrictive materials, a bias field (*B*_bias_) offsets the ac magnetic field to maximize magnetostriction and, thus, the piezoelectric response. (**C**) Equivalent circuit of an ME-OLED, with the ME transducer device represented by an ac current source and a capacitor in series due to the capacitive nature of piezoelectric materials. To ensure that current flows in both directions, two OLEDs are connected in antiparallel configuration. (**D**) Schematic illustration of an antiparallel device configuration (ac-OLED), with two OLED subcells deposited directly on top of each other. (**E**) Illustration of complete ME-OLED device consisting of an electrically insulated ME device that serves as substrate for the ac-OLED. The circuit is closed by connecting the intermediate OLED electrode with the back side of the ME transducer. A multilayer thin-film encapsulation protects the entire device. (**F**) Light emission from a wirelessly operated ME-OLED device placed on a plastic carrier. (**G**) Photograph of an ME-OLED demonstrator from the side, next to a two-Euro coin for reference in size.

The composite ME effect relies on the combination of two materials: a magnetostrictive material, which expands in the presence of magnetic fields, and a piezoelectric material, which generates a voltage in response to mechanical strain. In composites of these two materials, the expansion of the magnetostrictive material strains the piezoelectric material, which allows for the conversion of oscillating magnetic fields into alternating voltages via intermediate mechanical oscillations. Figure 1A shows the principle for a bilayer laminate operated in a longitudinal field.

To maximize the magnetostrictive strain for a given magnetic field, an additional bias magnetic field allows the operation of the device at the turning point of the magnetostriction curve. Furthermore, the laminate operates at its mechanical resonance frequency to maximize the induced strain and voltage (*33*, *34*). Figure 1B depicts the applied magnetic field, the resulting magnetostriction, and the sinusoidal voltage generated by the ME transducer.

We used a bilayer ME transducer consisting of the magnetostrictive material Metglas and the piezoelectric material lead zirconate-titanate (PZT) compounded by an epoxy. The device performance was evaluated using a custom-made characterization setup consisting of a pair of Helmholtz coils providing the dc bias field, a center coil providing the ac field, and a custom sample holder (fig. S1). On its own, i.e., before integration of an OLED, our bilayer ME transducer reached performance levels comparable to similar systems described in the literature (fig. S2) (*19*, *35*).

Because of the capacitive nature of piezoelectric materials, it is not sufficient to simply connect a single LED to the ME transducer. Instead, two OLEDs in an antiparallel configuration allow charges to flow in both directions (Fig. 1C). OLEDs are ideal for this as they can be stacked on top of each other during device fabrication, and their electrodes can be configured to provide the required antiparallel connection (Fig. 1D). In the past, such an ac-OLED configuration was investigated for applications in white light emission (*36*), automotive lighting (*37*), and most recently optogenetics (*38*). In using an ac-OLED on the ME transducer, we were able to harvest both half-cycles of the sinusoidal ME voltage for light-emission (Fig. 1B, bottom).

Figure 1E illustrates the final device structure, in which an ac-OLED is deposited directly onto the ME transducer. We chose to deposit the OLED on the Metglas side of the transducer as the short-wavelength component of its roughness is lower than for the PZT side (fig. S3). Because Metglas is conductive, parts of its outward-facing surface were isolated to ensure electrical separation of the different OLED electrodes. A conductive link connected the isolated electrode then to the other side of the ME device to close the circuit. Last, a robust multilayer thin-film encapsulation protected the entire structure ensuring its stability in air and aqueous solutions (*31*). The resulting devices showed bright and stable emission when placed inside the driver coil (Fig. 1F and movie S1). Their total thickness was well below 200 μm (Fig. 1G).

### OLED design optimization

**Fig. 2.**
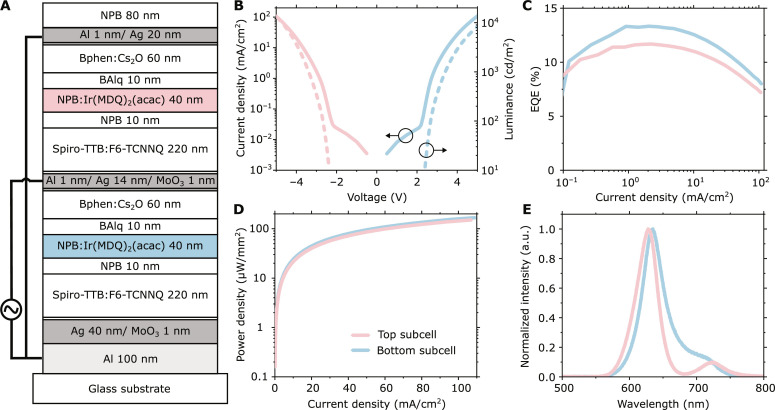
Architecture and characterization of ac-OLED. (**A**) Architecture of the ac-OLED stack, consisting of two second-order red phosphorescent subcells with the emissive layers of the bottom and top subcell depicted in blue and red, respectively. (**B**) Current density–voltage–luminance (JVL) characteristics of each subcell for an optimized ac-OLED, using the color code introduced in (A), with luminance shown as dashed lines. (**C**) Corresponding EQE for each subcell. (**D**) Optical power density emitted by the top and bottom subcell versus applied current density. EQE and optical power density data have been corrected for the non-Lambertian angular emission characteristics of each subcell (fig. S5). (**E**) Forward emission spectra of the bottom and top subcells. a.u., arbitrary units.

We chose a top-emitting ac-OLED stack with two red phosphorescent emissive layers based on Ir(MDQ)_2_(acac) placed in the second order maximum of the electric field of each subcell, as schematically shown in Fig. 2A. This allowed single-color emission and ensured high efficiency as well as high stability and excellent yield, even in the presence of a rough substrate (*28*, *39*). The transport layer thicknesses were optimized for maximum efficiency via optical transfer matrix modeling (fig. S4).

Figure 2 (B to D) summarizes the performance of the optimized device under dc operation and with a conventional wire connection. The top subcell reached a luminance of 13,400 cd/m^2^ at 5 V and an external quantum efficiency (EQE) of above 10%, and the bottom subcell reached 8300 cd/m^2^ and an EQE above 13%. The lower luminance but higher EQE of the bottom subcell is due to its super-Lambertian angular emission profile (fig. S5). At a current density of 100 mA/cm^2^, both subcells deliver optical power densities exceeding 150 μW/mm^2^. The forward electroluminescence spectra of both subcells show emission peaks at 627 and 635 nm, respectively (Fig. 2E).

The operational lifetime under dc operation was evaluated separately for each substack of the ac-OLED, using constant current driving at 25 mA/cm^2^, which corresponds to an initial luminance of about 4000 cd/m^2^ (fig. S6A), i.e., matching the average luminance observed for the ME-OLEDs under wireless operation (see below). The time until the luminance decayed to 70% of its initial value (LT_70_) was more than 1200 hours for the top subcell and more than 1900 hours for the bottom subcell. In addition, we performed a lifetime test under driving with a sinusoidal ac voltage (frequency 130 kHz; constant *V*_pp_ = 9 V, again corresponding to a mean initial luminance of 4000 cd/m^2^). These measurements showed no considerable degradation after more than 200 hours of operation (fig. S6B). In addition, the cyclic stability of the ME laminate might be an issue for long-term operation of our devices. Ma *et al.* (*40*), for instance, observed a degradation of ME performance after about 0.5 billion cycles at resonant conditions. However, for our ME transducers, which use a different material system, we did not observe any degradation after 5 hours of continuous operation at resonance conditions (143.4 kHz in this case) and with 2.3-mT dc and 1.5-mT peak ac magnetic fields; this corresponds to about 2.5 billion cycles (fig. S7).

### ME-OLED design and performance

**Fig. 3.**
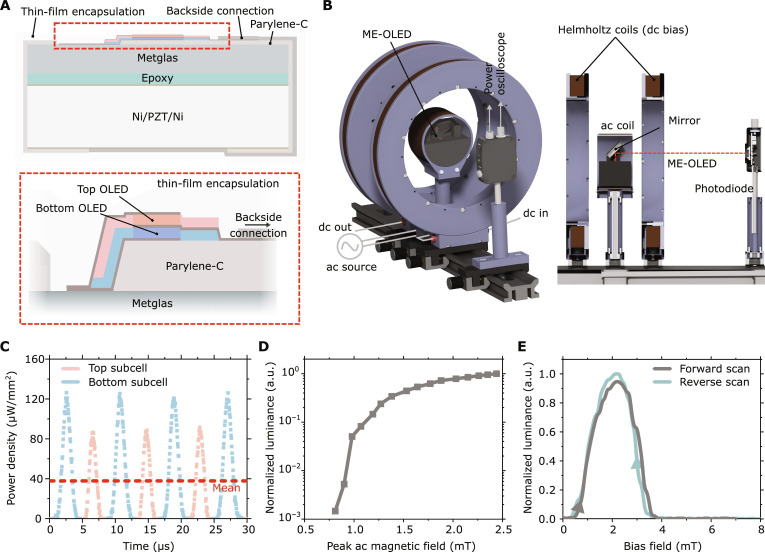
Design and characteristics of the ME-OLED. (**A**) Schematic illustration of ac-OLED integrated on an ME transducer. The Metglas surface is partially isolated with Parylene-C. The ac-OLED consisting of bottom (blue) and top (red) subcells is subsequently deposited over the Metglas and Parylene-C surface so that its intermediate electrode is electrically isolated from the top and bottom electrodes. A conductive link connects the intermediate electrode to the back surface of the ME transducer, and a thin-film encapsulation seals the entire device. (**B**) Characterization setup consisting of a pair of Helmholtz coils to provide a dc bias and a center ac magnetic field coil. For this measurement, the ME-OLED is placed in the center of the setup and a mirror is placed at a 45° angle to guide the light to a photodiode placed outside the region of strong magnetic field. (**C**) Evolution of optical power density generated by the ME-OLED over time, showing two distinct peaks corresponding to emission from the bottom (blue) and top (light red) subcells, and time averaged power density (red). Data corrected for the non-Lambertian angular emission characteristics of the device (fig. S9). (**D**) Luminance generated by the ME-OLED versus the magnitude of the ac magnetic field. (**E**) Luminance versus strength of bias field for a forward sweep (gray) and a reverse sweep (turquoise) and for a peak ac magnetic field of 2 mT.

Next, we deposited an ac-OLED with the optimized stack architecture and an active area of 2 mm by 2 mm directly onto an ME transducer introducing a patterned electrical isolation layer to ensure electrical separation of the intermediate electrode from the top and bottom electrodes (Fig. 3A). The devices were characterized using our custom-developed coil setup and a mirror guiding the emitted light to a calibrated photodiode placed outside the magnetic field (Fig. 3B). The time-resolved photodiode signal revealed two distinct series of emission peaks, each repeating at the frequency of the applied magnetic field (Fig. 3C). We attribute each series of peaks to emission from one of the two subcells of the ac-OLED. The brighter of the two subcells reached a peak optical power density of over 120 μW/mm^2^ under fully wireless operation, which corresponds to a peak luminance of more than 11,000 cd/m^2^ (fig. S8). The time-averaged power density generated by the ME-OLED was about 40 μW/mm^2^. Multiplying this number with the time of exposure yields the light dose, which is an important figure of merit for many potential applications of our devices, e.g., in optogenetics, photodynamic therapy and photobiomodulation (*41*–*43*).

In analogy to the luminance-voltage curves used to characterize conventional wired OLEDs, we analyzed the normalized luminance versus the applied ac magnetic field, which showed that our ME-OLEDs turn on at a peak ac magnetic field of about 0.8 mT (Fig. 3D). Furthermore, the optimum bias field for the ME-OLED was determined to be 2 mT (Fig. 3E).

### Addressing of separate ME-OLEDs by resonance tuning

**Fig. 4.**
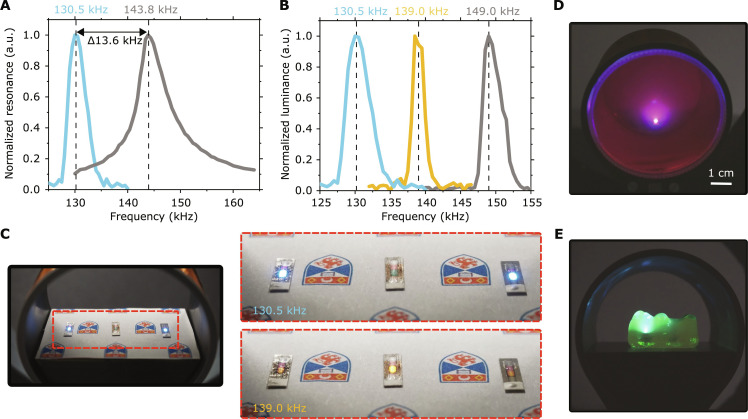
Resonance frequency based addressing of ME-OLEDs. (**A**) Normalized luminance versus frequency (blue) and normalized voltage versus frequency (gray) for a freely oscillating ME-OLED and an electrically contacted ME transducer, respectively. (**B**) Luminance versus frequency curves for three different ME-OLEDs with slightly different transducer sizes. (**C**) Photographs of one red and two blue ME-OLEDs that can be activated separately by tuning the ac magnetic field to their specific resonance frequencies. (**D**) Wirelessly operated blue ME-OLED embedded in gelatin stained with a red-emitting fluorescent dye. (**E**) Fluoresceine-stained gummy bear expanded by prior immersion in water, wirelessly illuminated by a blue ME-OLED positioned inside its head.

Figure 4A shows the luminance of a freely oscillating ME-OLED over the frequency of the driving ac magnetic field, and for reference also the voltage generated by a bare ME transducer without an OLED but with two external electrical contacts. Both traces show a clear resonance, with a strongly enhanced luminance and voltage at their respective resonance peak. The resonance in luminance for the complete ME-OLED is considerably narrower than the resonance in voltage measured for a bare ME transducer; this difference is due to the nonzero turn-on voltage of the OLED. Furthermore, the resonance frequency observed for the ME-OLED is considerably lower—by more than 13 kHz—than the voltage resonance of the bare ME transducer. We attribute this shift to a dampening of the oscillation of the bare ME transducer due to the fixed electrical contacts needed for the voltage measurement.

The frequency at which the resonance of an ME transducer occurs depends on its exact dimensions. This offers a simple route to the addressing of individual ME-OLEDs. To illustrate this, we slightly varied the size of the ME transducer, and in doing so, we obtained three devices with resonance frequencies at 130.5, 139.0, and 149.0 kHz (Fig. 4B). Because there is no overlap between the resonance curves of the three devices, each can be activated independently by tuning the ac magnetic field to the respective resonance frequency. To demonstrate this concept more clearly, in addition to the red-emitting ME-OLEDs described so far, we fabricated blue ME-OLEDs on the basis of the fluorescent emitter 2,5,8,11-tetra-*tert*-butylperylene (TBPe) using ME transducers with a resonance at 130.5 kHz (the device stack and its performance characteristics are shown in fig. S10). Without extensive optimization, we obtained peak and time averaged power densities of 200 and 50 μW/mm^2^, respectively, when operating the blue ME-OLEDs wirelessly. Figure 4C and movie S2 show the separate activation of two such blue ME-OLEDs at 130.5 kHz and of one red ME-OLED at 139.0 kHz, with all three devices placed inside the same coil.

For true multiplexing, only one ME-OLED should be on for a given frequency. The average frequency range over which the ME-OLEDs shown in Fig. 4B turn on is about 7 kHz. This means that ME-OLEDs of different resonances can be spaced by about 3.5 kHz to remain independently addressable. For a 100-kHz frequency range, for instance, a total of 28 devices can therefore be operated independently. For purely length-based tuning of the resonance frequency, this would require rectangular devices with lengths between 7.4 mm (200 kHz) and 14.9 mm (100 kHz). For higher frequency ranges, the length difference, however, is much more subtle. For instance, to cover the frequency band between 400 and 500 kHz, devices with lengths between 3.7 and 3 mm are required. In addition, the resonance frequency can be tuned by adjusting the shape of the ME transducer. Depending on the required brightness levels, further narrowing of the frequency response can be achieved by using lower-brightness operation. To illustrate the operation of our devices in an optically scattering or opaque medium, such as deep inside tissue, we placed blue-emitting ME-OLEDs inside a container filled with gelatin that was stained with the red fluorescent dye cresyl violet perchlorate. This allowed us to visualize the emission of the devices and their ability to photoexcite molecules in their surrounding (Fig. 4D). Operating an ME-OLED at a horizontal depth of 23 mm and a vertical depth of 33 mm inside the gelatin, we observed bright red fluorescence from the dye and some blue light waveguided to the outer edge of the gelatin-filled beaker. Figure 4E further illustrates the operation of ME-OLEDs deep inside a watery environment. Additional examples of wirelessly driven ME-OLEDs located deep inside tissue phantoms are shown in fig. S11.

We tested the sensitivity of our ME-OLEDs to changes in the used magnetic field (fig. S12). As the longitudinal component of the magnetic field changes as the cosine of the angle to the central axis of the coil, the luminance follows a similar behavior upon tilting the device. Likewise, when moving the ME-OLED along the central axis of the magnetic coil, the effective magnetic field will also decrease once the device comes close to the outside of the coil or leaves the volume of the coil altogether. In the case of the single coil used in this work, the luminance of our ME-OLEDs decreases to about half the initial luminance when leaving the coil, and the device turns off completely when it is about 17 mm away from the coil. However, the form of the decrease in magnetic field with distance to the coil center depends on the shape of the magnetic coil. Using a pair of Helmholtz coils to generate the ac magnetic field, for instance, one could generate a uniform field over a much larger volume.

## DISCUSSION

By providing an array of tools and treatment regimes, wireless light sources are set to revolutionize various aspects of display applications, neuroscience, and medicine. Wirelessly powered light sources promise to enable minimally invasive deep-tissue treatment as well as light-controlled experiments in freely behaving animal models. However, in many cases, the bulky form factor of existing devices and the substantial absorption of the transmitted energy by the surrounding biological tissue represent severe challenges. In this work, we introduced a concept for a compact wirelessly powered light source that operates by low-frequency magnetic fields that incur minimal absorption losses in many materials, including in biological tissue.

We developed and optimized an ac-OLED stack to realize the antiparallel diode configuration required for operation on an ME transducer. ac-OLEDs were optimized for efficiency, with both subcells exceeding 10% EQE. In addition, we developed a thin-film isolation protocol to allow direct deposition of the OLED stack onto the conductive ME transducer surface. The ability to stack OLEDs in an ac-OLED design and to deposit them directly on the ME transducer represents a fundamental advantage over inorganic LEDs that must be placed side by side and require a more involved and error-prone pick-and-place process. Our final, optimized, red-emitting ME-OLEDs provided about 40 μW/mm^2^ of optical power density under fully wireless operation. This value is sufficient to, for example, perform robust optogenetic stimulation when using modern optogenetic channels such as CsChrimson. Last, our fabrication process can be universally applied to different OLED designs as demonstrated by changing the emission color of the ac-OLEDs to blue.

Despite the high optical output power, the overall power transfer efficiency of the system is relatively low; about 3.2 W of continuous power are needed to maintain an ac magnetic field of 1.5-mT peak, which results in only 17.5 mW of average electrical power provided by the ME transducer, i.e., 0.5% power efficiency at 200-ohm load resistance. For a more realistic OLED load resistance of 5 kilohms, it is further reduced to about 0.16% power efficiency. To obtain the power efficiency from magnetic field generation to light generation, it further must be weighted by the ac-OLEDs power efficiency, which is on the order of 5%. The relatively low overall power efficiency of the system is not unexpected as the system has not been optimized for the efficiency of power transfer but for minimum size and ease of use. Reducing the ac coil size can considerably reduce the power required for the generation of a magnetic field of the same magnitude and thereby allow a more efficient use of the available flux density. Furthermore, more efficient ME transducer designs can be explored in the future to further enhance power efficiency.

Our work demonstrates some of the unique advantages of OLEDs as compared to inorganic LEDs, such as color tunability and the ability to directly fabricate them on different substrates. It is the latter property that is of most importance for this work because inorganic LEDs would have to be picked and then placed on the ME substrate in the right orientation using conductive glue or solder. Furthermore, this property allowed us to realize the required antiparallel two-diode configuration by stacking two translucent OLEDs on top of each other, which again considerably reduced the complexity of the fabrication process. This allows to circumvent the use of fundamentally more complex rectifying circuitry. It should be noted that using e.g., a full wave rectifier bridge, a single unit dc OLED stack with even higher performance could be used, potentially increasing the overall power efficiency of the wireless light source. However, this would come at the price of increased fabrication complexity and device size. While inorganic LEDs tend to provide higher power efficiency and better long-term stability compared to OLEDs, we note that state-of-the-art OLED encapsulation, such as the one used in this work, can mitigate the stability issue of OLEDs to a considerable degree.

To the best of our knowledge, our wireless light source is the smallest device reported for wireless operation deep inside optically scattering, watery environments to date. It has a total volume of 6.7 mm^3^ and a weight of less than 50 mg, most of which originates from the ME transducer, with the OLED and encapsulation combined adding only about 0.3 mg in weight. Being based mostly on vacuum processes, our fabrication protocol is scalable, and we expect that a further reduction in size by about one order of magnitude is achievable without fundamental changes to device design or fabrication process. We further expect the resonance frequency of these future devices to remain below 1 MHz, thus still ensuring low absorption of the supplied magnetic field in tissue and other materials.

As a direct result of their turn-on voltage, ME-OLEDs offer the advantage of being truly off when exposed to an off-resonance magnetic field. Furthermore, as different ME-OLEDs can be addressed by adjusting their size and hence resonance frequency, no additional on-device electronics will be needed for applications requiring the operation of multiple ME-OLEDs in independent clusters, such as simple displays or to expose different sites in an organism to specific optical treatment regimes. Potential applications for ME-OLEDs include optogenetic stimulation of freely moving animal models or intrabody photodynamic therapy. For these applications, the generation of milli-Tesla magnetic fields in the 100-kHz to 1-MHz regime remain an engineering challenge as these fields need to be present across a large volume. Initial applications for ME-OLEDs in the realm of optogenetic laboratory experiments might therefore focus on stationary stimulation where stimulation is only required when the animal approaches a certain area (e.g., drug addiction experiments). In the case of intrabody photodynamic therapy, larger animal models and ultimately human patients might be able to wear a lightweight coil setup for continuous stimulation. Temporary stimulation on the other hand could provide deeper tissue stimulation but would be limited to larger coil setups that generate magnetic fields of suitable strength across the whole body.

## MATERIALS AND METHODS

### ME device fabrication

Prepoled lead-zirconate titanate sheets (130-μm-thick PZT-5A, piezo.com, USA) were cut with a laser cutter (RDX500; Pulsar Photonics, Germany) into 24 mm by 24 mm pieces. A foil of an iron alloy containing silicon, boron, and manganese (Metglas 2605SA1; Metglas, USA) was cut into pieces of comparable size as the PZT using scissors. Two component adhesive (West System 105/206, USA) was spin coated onto the cut Metglas substrate at 3000 rpm for 120 s (EMS 6000; Electronic Micro Systems, UK) (*44*). Cut PZT pieces were bonded to the Metglas and cured at 55°C in a drying oven over several hours. Subsequently, the Metglas-PZT laminates were laser cut to the desired size.

### OLED device fabrication

OLEDs were fabricated via thermal evaporation at <1 × 10^−7^ torr base pressure in a vacuum chamber with multiple thermally controlled sources (EVOVAC; Angstrom Engineering, Canada). Quartz crystal oscillators were used to determine the deposited thickness. Three different shadow masks were used to deposit the bottom and top electrodes, organics, and intermediate electrode, respectively. The organic materials 2,2′,7,7′-tetra(*N*,*N*-di-p-tolyl)amino-9,9-spirobifluorene (Spiro-TTB), 2,2′-(perfluoronaphthalene-2,6-diylidene) dimalononitrile (F_6_TNAP), *N*,*N*′-bis (naphthalen-1-yl)-*N*,*N*′-bis (phenyl)-benzidine (NPB), bis (2-methyldibenzo-[f,h]quinoxaline)(acetylacetonate) iridium (III) (Ir (MDQ)_2_acac), bis (2-methyl-8-quinolinolate)-4-(phenylphenolato) aluminum (BAlq), 4,7-diphenyl-1,10-phenanthroline (BPhen), 2-methyl-9,10-bis (naphthalen-2-yl) anthracene (MADN), and TBPe were purchased from Lumtec (Taiwan) and used without further purification. The finished devices were transferred into a nitrogen-filled glovebox for encapsulation without exposure to air. Reference devices were fabricated on glass substrates (Eagle XG; Corning, USA) that were cleaned by subsequent ultrasound cleaning with acetone and isopropanol. Reference devices were encapsulated together with a getter material with cleaned cavity glass lids glued to the substrate using ultraviolet curable resin.

### ME-OLED device fabrication

The cut ME devices were isolated using a parylene deposition chamber (Parylene P8; Diener, Germany) via chemical vapor deposition. Briefly, 3 μm of Parylene-C (diX C; KISCO, Japan) was deposited on each side of the ME device to ensure edge encapsulation. We confirmed successful isolation by dipping the substrate into an ionic solution and using a multimeter, as depicted in fig. S13 (*45*). Surface electrodes on the Metglas and on the PZT side were exposed using inductively coupled plasma reactive ion etching (ICP-RIE; SI 500; Sentech, Germany) with 5-sccm SF_6_, and 50-sccm O_2_ flow at 300-W ICP and 30-W RF power for 12 min. Patterning was achieved using a custom aluminum holder with 0.2-mm-thick aluminum shadow masks. To prevent corrosion of the exposed Metglas during the oxygen plasma treatment, the etched devices were subsequently immersed in high concentration citric-acid solution for 15 min. Isolated devices were transferred to a thermal evaporation chamber for OLED deposition as described above, then transferred to a nitrogen-filled glovebox. To connect the intermediate OLED electrode to the exposed PZT surface electrode, devices were dip coated in silver ink (ACHESON 1415; Plano GmbH, Germany) using a custom-made dip coater that fits into the glovebox. After drying the silver ink, the devices were encapsulated using a thin-film encapsulation process consisting of two repeated stacks of 50-nm-thick alternating Al_2_O_3_/ZrO_2_ and 3-μm Parylene that were deposited via atomic-layer deposition (Savannah S200; Veeco, USA) and chemical vapor deposition, respectively, as described in more detail by Keum *et al.* (*31*).

### ME device characterization

ME devices were characterized using the coil setup depicted in fig. S1, which consists of a pair of Helmholtz coils providing dc offset and a centering ac coil for the ac magnetic field. Because of the strong impedance of the coil at high frequencies, a custom variable capacitor was attached to the ac coil and adjusted during operation to always hit the inductor-capacitor (LC) resonance of the circuit. A square signal of the desired frequency was generated by a digital clock generator (SI5351A; Silicon Labs, USA) and amplified by a custom class-D power amplifier built around a gate driver (IR2113; Infineon Technologies, Germany) in a half-bridge configuration. The magnitude of the ac magnetic field was controlled from 0- to about 3-mT peak by adjusting the voltage of a variable laboratory bench power supply (PPS-16005; Voltcraft, Germany). The magnitude of the dc field was controlled by adjusting the current of another variable laboratory bench power supply (3005P, Korad Technology, China). A custom sample holder with two spring pins was designed to contact the ME laminates without the need for any permanent wire connection (fig. S1). A pick-up coil on the sample holder was used to measure the applied ac field. The pick-up coil voltage as well as the ME response were measured using an oscilloscope (DS1000Z; Rigol, China). The clock generator, class-D amplifier, and variable capacitor were controlled using a microcontroller board (Arduino nano; Arduino, USA). All components were interfaced via custom python software to a measurement PC to facilitate automated measurements (DOI: 10.5281/zenodo.10546505).

### OLED characterization

The two OLED subcells of the ac-OLEDs were characterized separately, starting with the bottom subcell. The data were individually corrected for angular emission profiles with a goniometer setup, as described by Archer *et al.* (*46*), using an updated version of the control software (DOI: 10.5281/zenodo.10546513 and 10.5281/zenodo.10546515). Operational lifetime was measured under constant current operation (M6000; McScience, Korea). The operational lifetime under ac driving was measured by supplying a sinusoidal voltage with a fixed amplitude with a function generator (Arbitrary Waveform Generator 33220A; Agilent Technologies, USA).

### ME-OLED characterization

ME-OLEDs were characterized using the characterization setup depicted in Fig. 3B. To minimize interference of the magnetic field with the photodiode and vice versa, the light from the device was guided outside of the area of the magnetic field using a silver mirror. The photodiode signal was captured with an oscilloscope.

### Fluorescent dye experiments

A stock solution of 6% gelatin was prepared, heated to 90°C and vigorously stirred for 10 min. The stock (200 ml) was mixed with 1 ml of 0.5% cresyl violet perchlorate in EtOH and poured into a custom-made Plexiglas cylinder that closely fit the dimensions of the magnetic coil. In another experiment, 50 ml of gelatin stock was mixed with 10 μl of 1 μM fluorescein in 0.1 M NaOH and poured into silicon molds of different shapes. All containers were cooled to room temperature to allow the gelatin to cure. Transparent gummy bears (Haribo, Germany) were immersed in deionized water for 48 hours. Because of osmosis, the gummy bears expand in size while keeping their initial shape. Afterwards, the gummy bears were fluorescently stained by immersing them in a concentrated solution of 0.5% fluorescein in 0.1 M NaOH for 12 hours.

### Optical simulations

Optical simulations were based on the transfer matrix method as described by Furno *et al.* (*47*). The outcoupling efficiency of ac-OLEDs was iteratively improved by modifying the top and bottom cavities separately in the simulation, with up to eight iterations. The optical constants used in the simulation were measured by variable angle spectroscopic ellipsometry (M-2000DI; J.A. Woollam, USA) of thin films on silicon substrates and modeled via CompleteEASE software (J.A. Woollam, USA).
