# Supplementary material for: Wireless magnetoelectrically powered organic light-emitting diodes
Source: Sci Adv. 2024 Mar 6;10(10):eadm7613. doi: 10.1126/sciadv.adm7613 (PMC10917343; doi:10.1126/sciadv.adm7613)
Supplement: Supplementary file 1 — Figs. S1 to S13 Legends for movies S1 and S2 [file sciadv.adm7613_sm.pdf]

Science Advances
AAAS

Supplementary Materials for

Wireless magnetoelectrically powered organic light-emitting diodes

Julian F. Butscher *et al.*

Corresponding author: Malte C. Gather, malte.gather@uni-koeln.de

Sci. Adv. **10**, eadm7613 (2024)
DOI: 10.1126/sciadv.adm7613

The PDF file includes:

Figs. S1 to S13
Legends for movies S1 and S2

Other Supplementary Material for this manuscript includes the following:

Movies S1 and S2

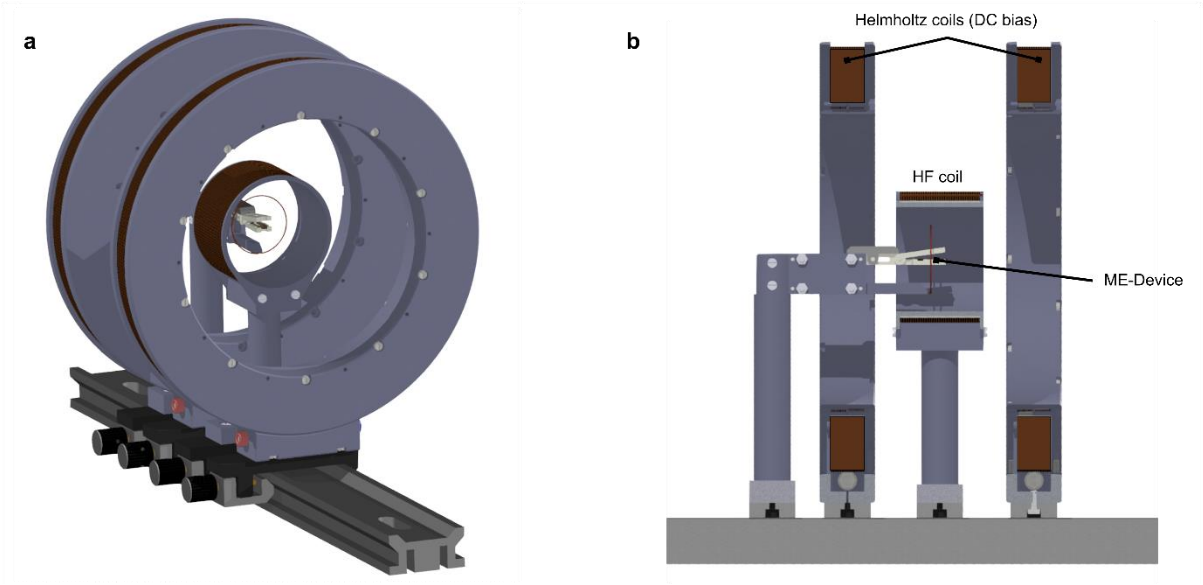

Fig. S1. Setup for the characterization of ME transducers. Magnetoelectric device characterization setup consisting of a pair of Helmholtz coils for the DC bias, a centering AC coil, and a custom ME device holder to make temporary contact to the surfaces of the ME laminate. a) Front view. b) Half-sectioned side view.

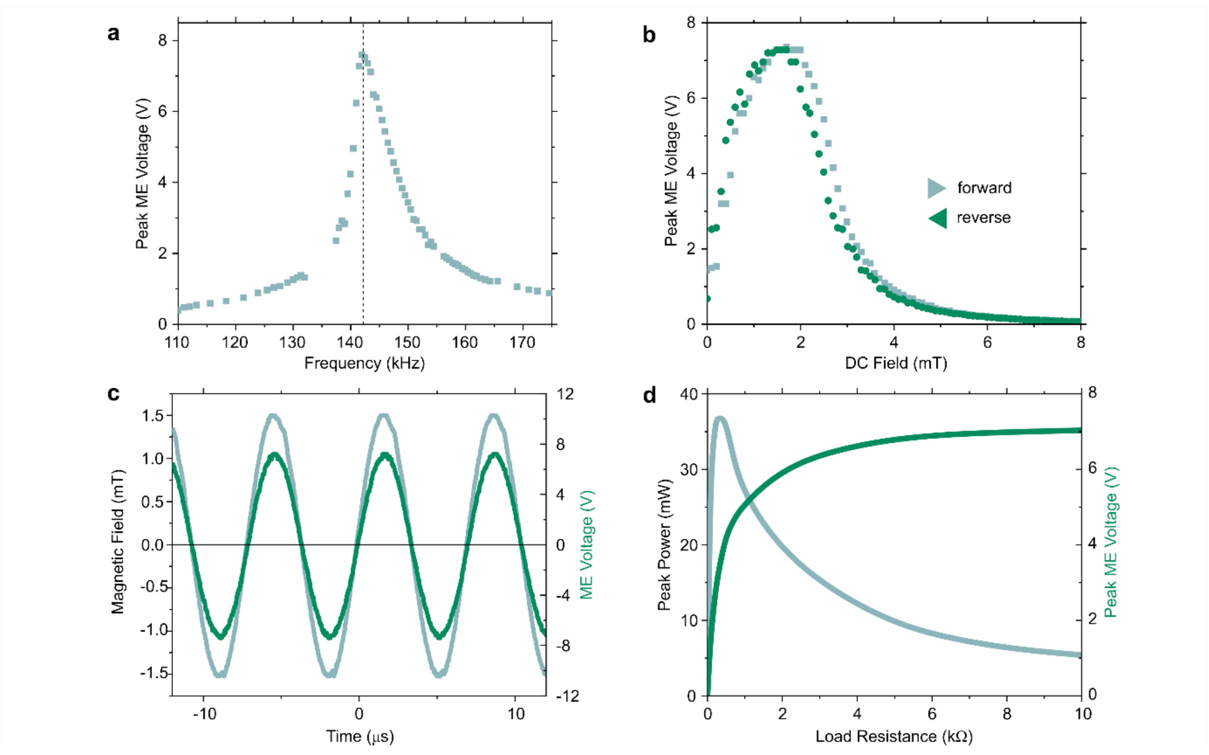

Fig. S2. Performance parameters of representative magnetoelectric laminate. The experimentally obtained performance characteristics of a representative device at 1.5 mT peak AC field measured with the setup depicted in Fig. 1. For a given system the longitudinal resonance frequency of a rectangular device depends on its length. We fabricated 11.45 mm x 3.45 mm long devices that show a) a mechanical resonance at around 142 kHz which we attribute to the first longitudinal vibrational resonance mode. At b) optimum bias field significant voltages of more than 7 V and d) an electrical power exceeding 35 mW at the maximum power point for an optimum load of 300 Ω are found. c) The voltage signal in the time domain oscillates at the frequency of the applied AC magnetic field.

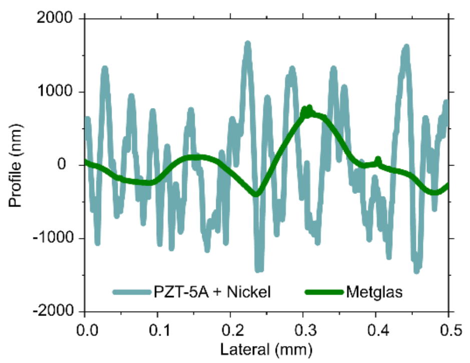

Fig. S3. Metglas and PZT surface roughness. Metglas and PZT roughness measured via profilometry measurements on a DektakXT (Bruker, USA) with a tip with radius 12.5 µm.

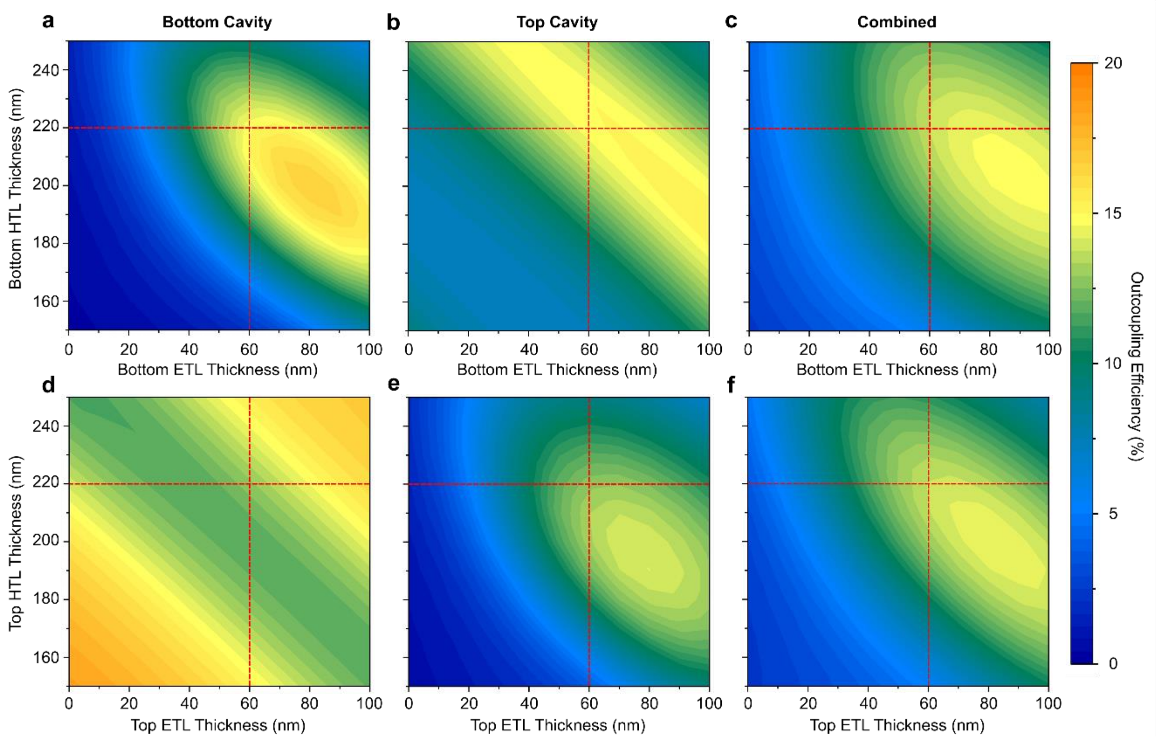

Fig. S4. **Transfer matrix simulations of the presented red AC-OLED stack.** Optical outcoupling efficiency of the two cavities depending on the transport layer thicknesses separately for a), d) the bottom cavity, b), e) the top cavity and c), f) both cavities combined. The red dotted lines indicate the thickness of the optimized OLED stack.

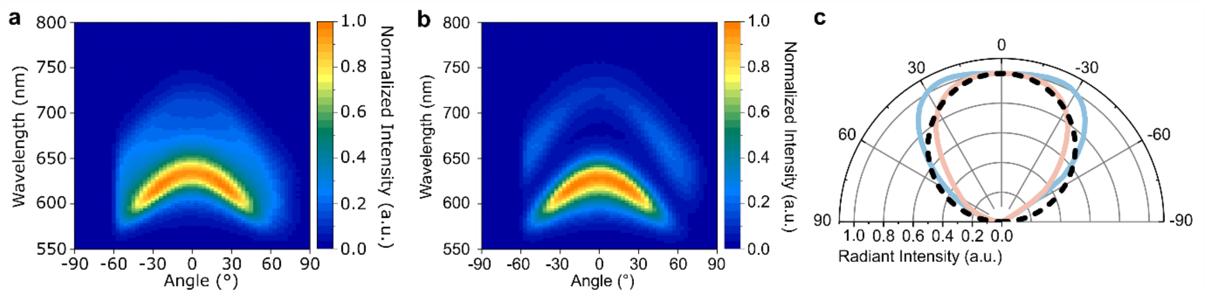

Fig. S5. Angular emission profile of optimized red AC-OLED on glass. a) Bottom and b) top stack and c) radiant intensity emission profile of bottom (blue) and top (red) stack as well as ideal Lambertian emission profile (black dotted). The spectra are cut for high negative angles due to the used sample holder as described in (46).

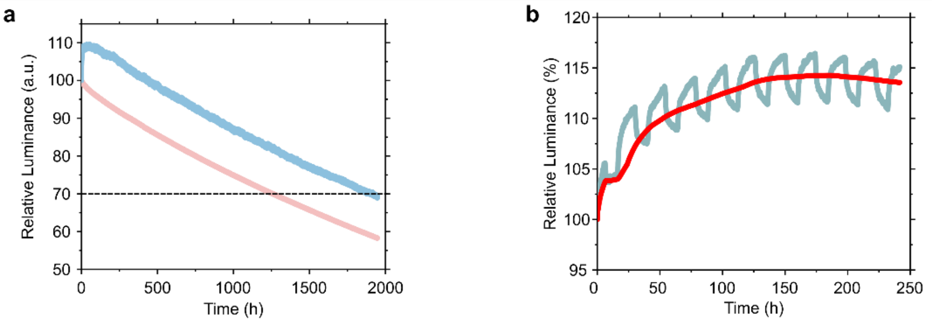

Fig. S6. Lifetime measurements of AC-OLED on glass. a) Lifetime of bottom and top OLED cavities under 25 mA/cm^2 DC constant current operation corresponding to an initial luminance of about 4000 cd/m^2. The dotted line represents the line of 70% initial luminance. b) Relative luminance under 130 kHz AC sinusoidal voltage operation and constant voltage bias of 9 V peak-to-peak, also corresponding to about 4000 cd/m^2 average initial luminance. The fluctuations in luminance originate from temperature variations within the measurement room and repeat with a 24 h periodicity. A 24 h running average is plotted in red to better indicate the development of the curve. The increase in luminance can most likely be attributed to the improvements for the bottom cavity as observed in a. This is likely due to an initial charge imbalance that is partly compensated with time.

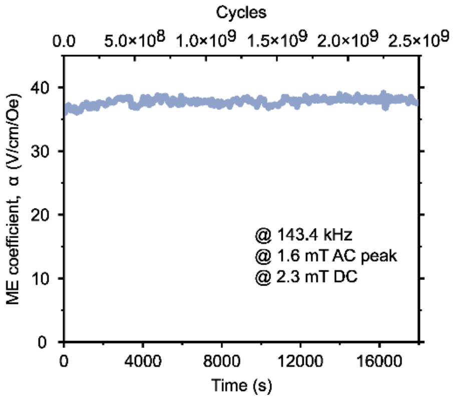

Fig. S7. Cyclic stability of an ME transducer at resonance. Device stability of a bare ME device under 5 hours continuous operation at its resonance frequency of 143.4 kHz and a peak AC field of 1.6 mT.

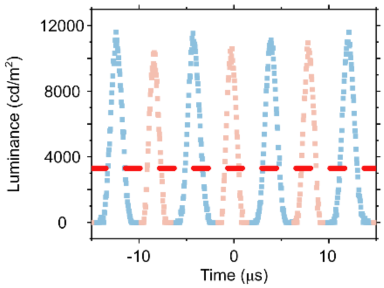

Fig. S8. Red ME-OLED luminance in time domain. ME-OLED luminance of wirelessly operated red device in time domain showing the distinct peaks corresponding to bottom and top OLED cavity.

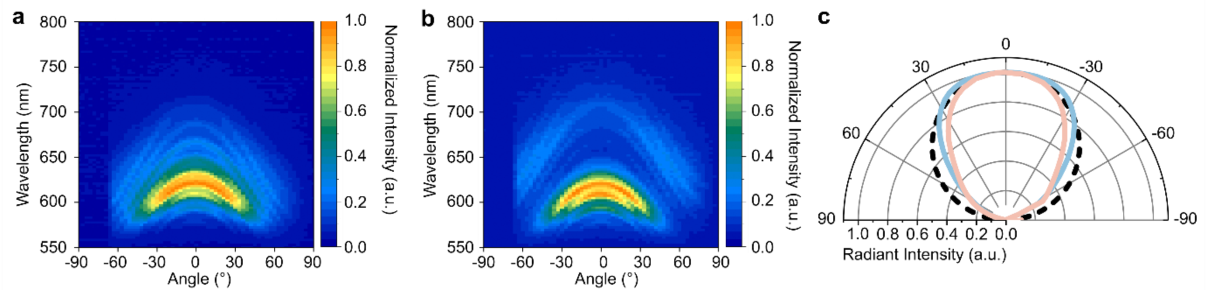

Fig. S9. Angular emission characteristics of electrically driven red AC-OLED on ME device. To ensure correct evaluation of the data, we used one ME-OLED per batch as a reference device and investigated its angular emission characteristics electrically. Shown are the measured angle resolved spectra of this device under wired operation with DC bias, a) for the bottom stack, b) for the top stack. The ripples in the AR spectra are attributed to thin film interference due to the encapsulation structure used. c) Wavelength integrated radiant intensity of both stacks (red top sub-cell, blue bottom sub-cell).

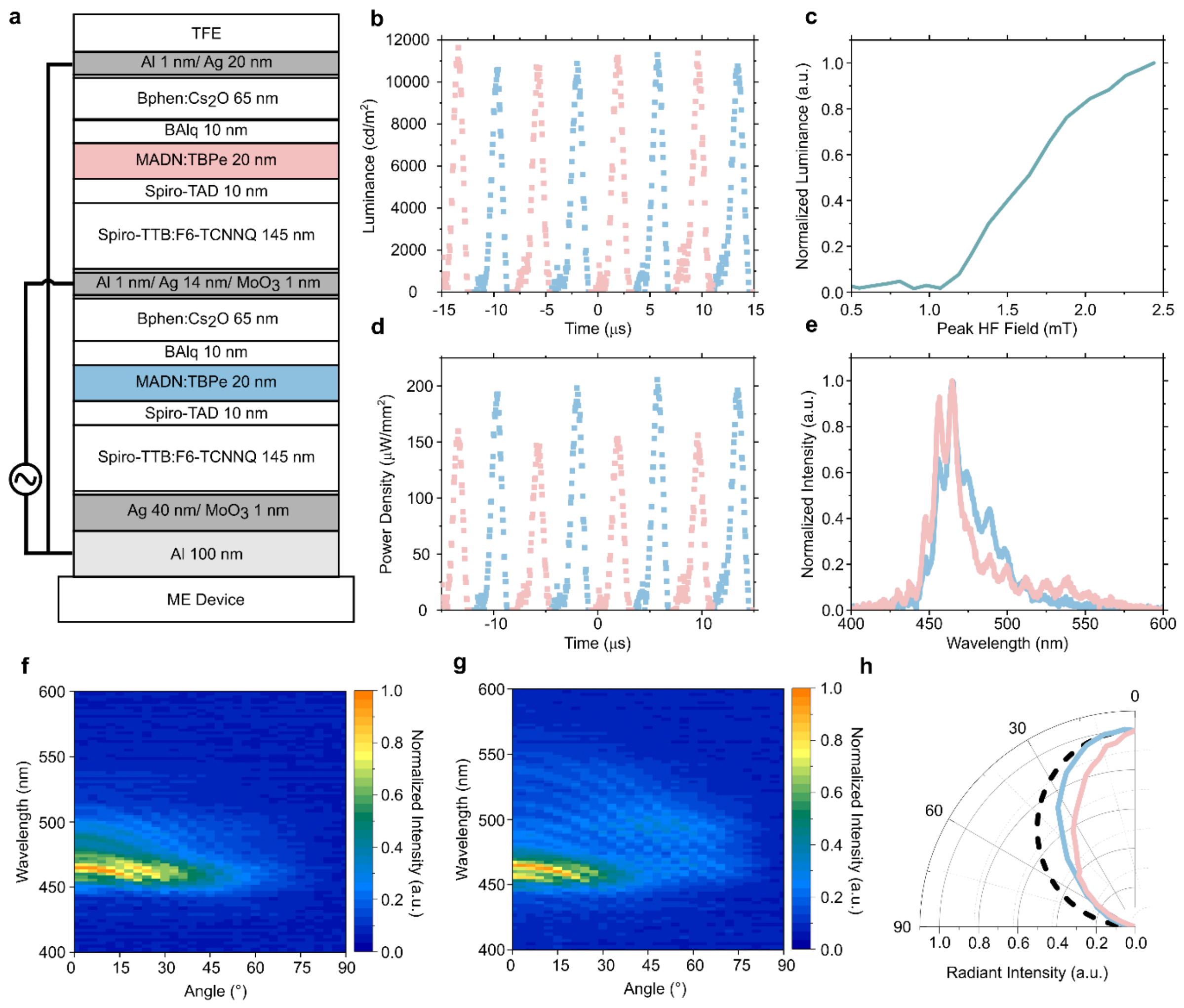

Fig. S10. Blue ME-OLED design and performance. a) Blue AC-OLED stack design. b) Time resolved luminance. c) Luminance field characteristics. d) Time-resolved power density. e) Forward emission spectrum. f) Angle resolved spectrum of bottom stack. g) Angle resolved spectrum of top stack. h) radiant intensity for different angles from 0° to 90°.

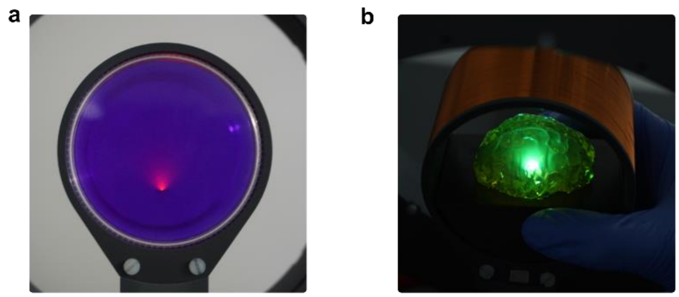

Fig. S11. Deep wireless operation of ME-OLEDs in fluorescently stained gelatin. a) Red ME-OLED in beaker with cresyl-violet stained gelatin. b) Fluorescein-stained gelatin tissue phantom molded into a half brain structure, wirelessly illuminated by blue ME-OLED underneath to excite green fluorescence.

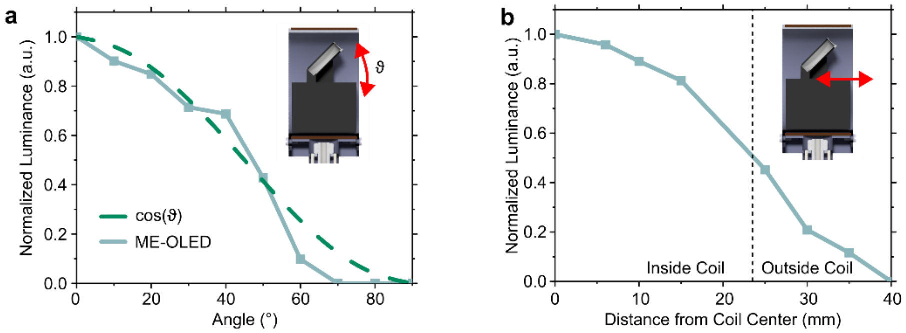

Fig. S12. Alignment characteristics of ME-OLEDs. a) At an angle with respect to the rotational axis of symmetry of the magnetic coil and b) a distance from the center of the AC coil.

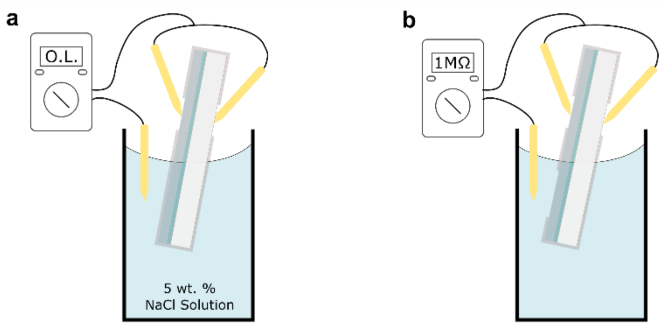

Fig. S13. Assessment of isolation of magnetoelectric laminates. After complete isolation, parts of the conductive surfaces were exposed again to contact them with a multimeter probe. By dipping the isolated part in a 5 wt.% NaCl solution and placing the second multimeter probe into the solution (45). Information on the quality of isolation can be obtained from the resistance measured. a) In case of perfect isolation, the multimeter shows no measurable resistance (open loop). b) In case of gaps in the isolation, the multimeter will show a high but measurable resistance value.

Movie S1.
Demonstration of ME-OLED operation

Movie S2.
Demonstration of separate activation of ME-OLEDs by resonance frequency tuning
